# Current Trends in Volume and Surgical Outcomes in Gastric Cancer

**DOI:** 10.3390/jcm12072708

**Published:** 2023-04-04

**Authors:** Luigi Marano, Luigi Verre, Ludovico Carbone, Gianmario Edoardo Poto, Daniele Fusario, Dario Francesco Venezia, Natale Calomino, Karolina Kaźmierczak-Siedlecka, Karol Polom, Daniele Marrelli, Franco Roviello, Johnn Henry Herrera Kok, Yogesh Vashist

**Affiliations:** 1 Department of Medicine, Surgery and Neuroscience, University of Siena, 53100 Siena, Italy; 2 Department of Medical Laboratory Diagnostics-Fahrenheit Biobank BBMRI.pl, Medical University of Gdansk, 80-308 Gdańsk, Poland; 3 Department of Surgical Oncology, Medical University of Gdansk, 80-308 Gdańsk, Poland; 4 Department of General and Digestive Surgery, Complejo Asistencial Universitario de León, 24071 León, Spain; 5 Organ Transplant Center of Excellence, King Faisal Specialist Hospital and Research Center, Riyadh 11211, Saudi Arabia

**Keywords:** gastric cancer, gastrectomy, hospital volume, surgical volume, centralization, textbook outcome, quality of care, healthcare

## Abstract

Gastric cancer is ranked as the fifth most frequently diagnosed type of cancer. Complete resection with adequate lymphadenectomy represents the goal of treatment with curative intent. Quality assurance is a crucial factor in the evaluation of oncological surgical care, and centralization of healthcare in referral hospitals has been proposed in several countries. However, an international agreement about the setting of “*high-volume hospitals*” as well as “*minimum volume standards*” has not yet been clearly established. Despite the clear postoperative mortality benefits that have been described for gastric cancer surgery conducted by high-volume surgeons in high-volume hospitals, many authors have highlighted the limitations of a non-composite variable to define the ideal postoperative period. The textbook outcome represents a multidimensional measure assessing the quality of care for cancer patients. Transparent and easily available hospital data will increase patients’ awareness, providing suitable elements for a more informed hospital choice.

## 1. State of Art

Gastric cancer represents one of the main causes of cancer mortality worldwide [1]. Although significant advances in diagnostic and therapeutic tools have improved survival outcomes, surgery remains the only curative therapy for gastric cancer patients. Surgical resection of the primary tumor with adequate lymphadenectomy is considered the only curative therapeutic approach for resectable gastric cancer, while preoperative and adjuvant chemotherapies may improve the outcomes aiming at the reduction of recurrence rate and the increase in survival [2,3]. 

However, the extension of lymphadenectomy is still an open issue between European and Japanese surgical schools [4]. At present, based on scientific and technical outcomes, the Western perspective on lymphadenectomy in gastric cancer surgery has been reversed. Consequently, most national and international scientific societies agree on D2 lymphadenectomy as the standard of treatment with curative intent [5]. Overall, the main goal of gastric cancer surgery is to improve patients’ postoperative recovery, resulting in a better quality of life, and to maximize long-term oncological outcomes through a proper surgical approach with a tailored lymphadenectomy [6,7]. 

Many novel gastric cancer classifications aimed at clinical and prognostic applications have been recently suggested [8]. The new classifications are based on tumor location, histopathology, gene expression, gene amplification, DNA methylation, several cancer-relevant aberrations, and oncogenic pathways [9,10,11,12,13,14]. The Cancer Genome Atlas (TCGA) and Asian Cancer Research Group (ACRG) [9,10] have proposed a molecular-based classification of gastric cancers finding new ways to treat the disease with a more personalized approach. Several reports highlighted specific demographic and pathological features (such as age, tumor location, invasion, and stage) shown by distinct molecular subgroups [9,10,15,16,17,18,19]. Similarly, the project High-tech Omics-based Patient Evaluation (HOPE) has established an updated molecular classification that predicts disease-specific and overall survival in patients undergoing radical gastrectomy [20].

Notwithstanding, despite advancements in surgical techniques [21,22], active involvement in clinical, translational, and basic research together with the improvements in perioperative care, short- and long-term outcomes still vary considerably among different providers and countries [23,24,25,26]. In an effort to reduce these variations and pursue the provision of high-quality cancer care, volume-based referral has been advocated as an adequate predictor for good quality of care [27]. In 1979, Luft HS et al. [28] introduced the concept of “*surgical volume*” stating that high-volume hospitals have better outcomes than low-volume hospitals for complex surgical procedures. 

## 2. Centralization

*“Centralization*” is defined as a process of concentration of resources, including staff, materials, infrastructures, knowledge, research, and expertise to enhance the quality of care achieving better financial efficiency. The centralization of major cancer surgery in hospitals with a high annual volume of procedures significantly reduces the risk of perioperative morbidity and mortality [29,30,31]. As a result, a plethora of research papers have investigated the relationship between surgical volume and outcome, and several policy strategies, particularly those designed to limit complex surgery to certified high-volume hospital and/or surgeons, have been debated. In 1999, the US National Cancer Policy Board of the Institute of Medicine published a statement to “*ensure that patients undergoing procedures that are technically difficult to perform and have been associated with greater mortality in lower-volume settings receive care at facilities with extensive experience*” [32]. 

Therefore, between the 1990s and 2000s, there was a shift also in private practice, such as the Leapfrog Group, for referrals being based on hospital volume [33]. Given these assumptions, some authors have recommended the creation of minimum volume thresholds to limit the number of centers with low levels of activity [34,35]. In 2008, Bilimoria KY et al. analyzed the distribution of 27,420 gastrectomies collected in the US National Cancer Database, identifying the lowest volume hospitals as those performing less than four and highest volume centers when performing more than seventeen gastric resections per year [36]. It was estimated that 179 perioperative deaths and 493 long-term deaths could have been avoided in high-volume centers, showing a higher risk of perioperative death and a worse 5-year survival for patients treated in low-volume hospitals [36,37].

Quality assurance has been increasingly recognized as a critical factor in the oncological surgical care process and, also for gastric cancer surgery, these associations between volume and outcome have been described [23,24,26]. In 2001, the Association of Upper Gastrointestinal Surgeons of Great Britain and Ireland (AUGIS) set the ideal threshold of volume standards for gastric cancer surgery at a minimum of 15–20 resections per year [38]. Subsequently, in 2003, research from Denmark highlighted the strong relationship between volume and outcomes, reporting less anastomotic leakages, a decreased 30-day mortality, and improved lymph node harvesting after the centralization of cases [38,39]. However, the cut-off point for the minimum number of surgical procedures was not exactly defined. On the other hand, several North American studies have reported conflicting results [31,40,41,42,43]. Past definition of high-volume center referred to a cut-off between 15 and 35 annual cases [31,40,41,43,44,45], whereas a recent international panel [46] defined consensus guidelines on the standard of care for gastric cancer surgery, setting the appropriate threshold for high-volume centers at more than 15 gastrectomies per year. In the Netherlands, the Dutch Health Care Inspectorate imposed a minimum of 10 gastrectomies per institution per year in 2012, and 20 per year from 2013. As a result, the total number of institutions performing gastric cancer surgery decreased, and the annual procedural volume per high-volume hospital increased [30,47]. In Italy, a minimum of 20 cases is considered the cut-off for referral centers treating gastric cancer. In 2017, a systematic evaluation of Italian hospital data, covering the years 2012 to 2015, identifies 40 cases per hospital as the cut-off for a relevant decrease in mortality [48]. These data need to be interpreted with caution because, according to this threshold, only 10.7% of total gastrectomies were performed in high-volume centers.

Even though the centralization of complex surgical oncology into high-volume hospitals has been prompted globally [38,49,50,51], an international agreement about the clear identification of high-volume hospitals as well as minimum volume standards has not yet been established. No study was able to identify specific thresholds on which outcomes change clearly and causally., and volume thresholds are usually set arbitrarily. 

Centralization is important for surgeons to gain sufficient experience and proficiency in order to develop their expertise and achieve high-quality surgery [52]. Most studies about trends in volume and surgical outcomes have assessed mortality as the primary indicator, suggesting that this variable has a positive association with the length of hospital stay [53], recovery time [54], cost of the hospitalization [55], related morbidity [56,57] and disease-free survival [58,59]. However, mortality alone, investigated through a simple logistic model, may be insufficient to establish surgical activity thresholds. or to encourage potential modification of organizational structures [60]. A regression that does not control for organizational effectiveness will find a positive relationship between volume–outcome, whereas it is organizational skills and proven internal protocols, not higher hospital volume, that drives improved patient outcomes [61]. The opportunity of having standardized clinical pathways and healthcare professionals perfectly integrated into the tumor board, such as digestive endoscopy, trained anesthetists, and interventional radiology, guarantees the optimization of the perioperative process and a timely and effective management of postoperative complications [62,63,64].

Another interesting issue is that health planning aimed at the centralization of rare diseases may increase the probability that patients will be treated in hospitals with a comprehensive range of experienced specialists (nursing, radiology, pathology, and geriatrics), services to support the provision of care (physiotherapy, dietetics, and psychosocial support) and free access to new technological advances [65,66]. Over the past decades, minimally invasive gastrectomy has become increasingly utilized, as lower complication rates and shorter hospital stays have been described, despite similar long-term survival [67,68,69]. Robotic-assisted gastrectomy might overcome some challenges, by offering improved visualization through 3D images and increased magnification, instrument articulation, superior ergonomics, and tremor filtration. Minimally invasive surgery has been demonstrated to be safe and effective, mainly if performed in referral centers, even if further trials are required to establish the superiority of robotic gastrectomy on long-term outcomes [70]. On a population level, the introduction of robotics is expected to have contributed to the centralization of cases in an unintended but potentially beneficial way. To date, Italy boasts more than 100 da Vinci surgical robotic systems, most of them from northern regions with an unequal distribution across the country. On the other hand, its true impact on cancer control, functional outcomes, and access to care is still opaque. Potential risks are longer waiting times from referral to surgery to having the surgical procedure and increased medical tourism [71]. New robotic systems are currently being developed, which will make surgical technologies more widely available, facilitate collaboration among surgeons, who may be separated by distance, in real-time, and decrease patient travel. On a professional level, recent evolutions in care, such as remote surgery, requires continuous training, creation, and revision of specific guidelines and protocols, bringing new challenges to surgical equipment and to their work [72].

Overall, the evidence from clinical data to support the advantages of centralization has not been proven beyond any doubt, showing previous studies on the “*gastrectomy case volume*” conflicting and heterogeneous results [73,74,75,76,77]. It is gradually becoming clear that a mere concentration of the number of cases per hospital or per surgeon is not enough.

## 3. Predictors for Good Quality of Care

In Europe, the mortality rate after gastric cancer surgery ranges from 2% in specialized centers [78] to 10% in certain nationwide registries [26]. Quality assurance has been regarded as the current main challenge for surgeons [27], in order to pursue the so-called “*rescue phenomenon”*, i.e., the ability to prevent minor postoperative events from developing into severe complications and death. 

Standardized surgical therapy is supported in surgical oncology, due to the weak evidence of the surgical randomized control trials, especially those focusing on chemotherapy. Many international initiatives, such as the new platform SURGCARE, a collaborative project between the European Society of Surgical Oncology (ESSO) and the Japanese Clinical Oncology Group (JCOG) [79,80], invested their resources and promoted quality assurance. In gastric cancer, the pursuit of evidence-based medicine and the shift toward precision surgery [81] have advocated the standardization of gastric cancer treatment and the creation of a standard level of competence. This application includes multimodal aspects of treatment, surgical competence with particular attention to the application of minimally invasive approaches, the establishment of a registry of complications as well as a medical database including follow-up [82]. 

For this purpose, the risk-adjusted and case mix-adjusted American College of Surgeons National Surgical Quality Improvement Program (ACS-NSQIP) has been established, with the aim to collect data that provide an accurate, correct, and thorough analysis, in order to help surgeons and hospitals to better understand the quality of their care than similar hospitals with similar patients [83]. Each hospital assigns a trained Surgical Clinical Reviewer to collect 30-day perioperative data on a web-based platform. Blinded information is shared with participant hospitals, allowing them to nationally benchmark their complication rates and surgical outcomes [84].

Over the past years, several studies have investigated the effect of hospital volume on gastric cancer surgery outcomes, leading to the concept that centralization results in better outcomes, acting as a proxy measure for various processes and providing the advantages of a qualified multidisciplinary team and a comprehensive multidimensional assessment [85,86], easier access to sophisticated cancer imaging equipment, availability of skilled surgeons, and better postoperative care facilities [30,87,88,89,90]. In this regard, an experienced radiologist with dedicated skills in gastric cancer metastasis detection (i.e., gastric carcinomatosis) is fundamental to allow for better patient selection [91]. Similarly, it has been proven that intensive care units (ICUs) with dedicated board-certified staff are associated with a lower post-gastrectomy mortality rate [92,93]. Additionally, early diagnosis as well as successful and effective management of postoperative complications might be better in high-volume hospitals [94]. Moreover, in an attempt to guarantee high-quality oncologic care, the discussion of clinical cases within a regional multidisciplinary expert panel is advocated [95]. 

In addition, the existing research does not focus on the patients-perceived quality of care [96]. A Swedish analysis emphasized that patient satisfaction arises from well-functioning care pathways, individualized care plans, continuity of treatment with local providers, accessibility for contact and information, involvement in the care process, and limited waiting time. A dramatic disadvantage of centralization is an increase in travel demands. A recent experiment conducted in England highlighted that patients were prepared to travel an average of 75 min longer to decrease their risk of complications by 1%, and over 5 h longer to reduce the risk of death by 1%, in line with the centralization trend [97]. Additionally, centralization should address real-life issues, such as postoperative continuity of care, long-term follow-up, and the possible need for urgent readmission [98]. The literature data suggested that most patients were prepared to travel long distances to receive specific care, but information on clinical outcomes of different hospitals is not widely available for the patients. 

The present finding raises the possibility to shift from “*output*” (maximizing the number of “*stuff*” produced and of tasks in the guidelines), to “*outcomes*” mindsets (applying to understanding your patients’ needs and solving their clinical problems). A clear focus on outcomes helps organizations succeed better by achieving “*patient centricity*” and maximizing the bottom line in terms of efficiency and costs. However, an organization that focuses primarily on solving its own problems (*“impact”*), will lose sight of its patients. Considering such evidence, it is mandatory to detect adequate predictors for good quality of care.

### 3.1. Hospital Volume

Despite the lack of unanimity [73,99], there is a growing recognition that multidisciplinary care in high hospital volume can improve postoperative mortality for gastrectomy [51,100,101]. 

Nelen SD et al. [77] reported a study aimed at investigating the outcomes of 250 gastric cancer patients after the centralization of surgery in the Netherlands since the introduction of the centralization policy in 2012. The treatment in high-volume hospitals resulted in an improvement in the percentage of patients treated with appropriate lymphadenectomy (21% vs. 93%, respectively), and a successful introduction of laparoscopic gastrectomies (6% vs. 40%, respectively). However, centralization did not realize an improvement in 30-day mortality as well as complication requiring a reintervention. More recently, the same Dutch study group reported the impact of centralization of gastric cancer surgery in a population-based setting. In this updated study comparing 3777 gastric cancer patients treated between 2009–2011 and 3427 between 2013–2015, the impact of the centralization was more evident in terms of improvement in surgical outcomes (lymph node retrieval and R0 resection rate), lower postoperative mortality and increased overall survival for all gastric cancer patients [102]. 

On the other hand, Claassen YHM et al. [39] did not report differences in morbidity and mortality rates between the hospital volume categories, ranked as very low (1–10 gastrectomies/year), low (11–20), medium (21–30), and high (31 or more). They postulated that patients referring to medium and high-volume centers had major comorbidities (co-morbidity score ≥3) or more frequently underwent total gastrectomy surgery. Moreover, a retrospective review of the CRITICS trial reclassified hospitals as low-volume (1–20 gastrectomies/year) and high-volume (21 or more) finding higher overall survival and disease-free survival from high-volume hospitals [103].

Agnes A et al. argued that the high-volume status is referred to surgeons performing a high number of gastric resections and to other measurable and non-measurable variables, such as case mix (complexity of operation, comorbidities), well-organized perioperative process (ICU, trained anesthesiologist, radiologist, and nurses, availability of other specialists around the clock), timely management of postoperative complications (continuous assistance from experienced physicians, interventional radiology, digestive endoscopy) and appropriateness of the indication resulting from multidisciplinary cancer boards [104]. Most of these aspects could directly improve early postoperative outcomes and influence failure to rescue phenomenon [105]. 

The UK National Esophago-Gastric Cancer Audit registered a 90-day mortality of <5% and an anastomotic leakage rate of 6.3% in gastric cancer surgery. Moreover, after adjustment, lower 30-day mortality and anastomotic leak rate were observed in hospitals with higher volumes, while higher surgeon volume was associated with a lower anastomotic leak rate [106]. A German observational study revealed that treatment in a very high volume is associated with lower in-hospital mortality compared to low-volume hospitals [107]. Similar results arose from the Taiwan National Insurance Research Database [108]. Interestingly, postoperative mortality was low for each hospital volume category in a retrospective French study [109] that reported the impact of institution volume on 90-day postoperative mortality after gastric cancer surgery. Postoperative mortality rate ranged from 4.3 to 10.2% and resulted in 7.9% in very high-volume hospitals (at least 60 resections/year). Those data suggest the role of other factors, such as hospital facilities, or timely recognition of complications, in determining outcomes [30]. It could be argued that death or complication after surgery are imperfect measures of surgical quality. 

On the other hand, a Japanese perspective on a total of 145,523 patients who underwent distal gastrectomy for gastric cancer by 11,914 surgeons at 2182 institutions has been recently published [110]. Hospital volumes were divided into 3 tertiles (low, 1–22 cases per year; medium, 23–51 and high, 52–404): An inversely proportional relationship between mortality rate and hospital volume was registered, resulting in the operative mortality of 1.9% in low-volume hospitals, 1.0% in medium and 0.5% in high (*p* < 0.001). Similarly, surgical complications such as anastomotic leakage, pneumonia, and surgical site infection were significantly higher in low-volume hospitals (*p* < 0.001) [110,111]. The same group recently analyzed a cohort of 71,307 patients undergoing total gastrectomy at 2051 institutions. Hospital volumes were divided into three tertiles: low, 0–11 cases per year; medium, 12–26, and high, 27–146. The peri-operative mortality rate passed from 3.1% in low-volume hospitals to 1.7% and 1.2% in medium and high volumes, respectively (*p* < 0.001). Surprisingly, the anastomotic leakage rate was not significantly different between low- and high-volume hospitals, while the rate of septic shock and medical complications of the nervous system were significantly higher in low-volume hospitals (*p* < 0.001) [112].

However, if Persi Diaconis and Frederick Mosteller’s “*law of truly large numbers*” was true, with a sufficiently large number of samples, any highly implausible result would be likely to be observed. Since the occurrence of probable events is never surprising, we highlight fewer probable events [113]. 

A South Korean study, using National Health Insurance Service (NHIS) Sampling Cohort data during 2004–2013, noted that if mortality decreased with increasing hospital volume, the risk of mortality increased again after reaching some level of surgery volume [35]. 

Another interesting topic is the assessment of procedure volume effect on patient outcomes after the perioperative period. Long-term outcomes could be strongly influenced by the appropriateness of patient selection for peri-operative therapies, the type of surgery, the technical skills of the surgeon, and the availability of a specialized pathologist to appropriate stage the disease. To date, only a limited number of studies investigating the relationship between hospital volume and long-term survival after gastrectomy have been published, with scarce and conflicting results [43,51,73,99,102]. Birkmeyer JD et al. [31] explored the relationship between hospital volume and late survival after different types of cancer resections, using the national Surveillance Epidemiology and End Results (SEER)–Medicare-linked database. They found a statistically significant association between 5-year survival and hospital volume, reporting a lower survival rate in low-volume compared with high-volume centers (25.6% vs. 32.0%, respectively), irrespective of differences in the use of adjuvant radiation and chemotherapy [31]. On the contrary, a prospective, population-based study of 3293 consecutive patients with esophageal or gastric cancer endorsed by the Scottish Audit of Gastric and Oesophageal Cancer (SAGOC) failed to demonstrate any correlation between hospital volume and postoperative morbidity or mortality, nor between survival and volume of patients neither for the hospital of diagnosis nor hospital of surgery [73]. 

There is much debate if positive relationship volume–outcome results from a practice-makes-perfect or a selective-referral mechanism. Under the first hypothesis, repeatedly performing procedures yields experience and enhances the organization of the surgical team, improving future outcomes. Under the second hypothesis, better outcomes attract more patients. Of course, practice-makes-perfect supports centralization, whereas selective-referral does not.

### 3.2. Surgeon Volume

The hospital volume and outcome relationship does not maintain its correlation at the individual surgeon level. As for hospital volume, similar attention was paid to the relationship between mortality rate and surgeon volume. Several reports have demonstrated an impact of surgeon activity on postoperative short- as well as long-term outcomes among patients undergoing gastric cancer surgery [110,114,115]. Even though 10-15 gastrectomies per year were suggested as a minimum surgeon volume for gastrectomy, [50,116], further evaluation in a large-scale cohort is needed [110].

Furthermore, it is hard to apply the same caseload threshold to clinical practice in different countries since the differences in epidemiology, biology, and treatment strategy can influence the cut-off value. 

In the Western setting, the lower incidence of gastric cancer also resulted in a lower average volume, which ultimately led to poorer opportunities for surgical trainees. In terms of postoperative results, the learning curve is considered optimized once the minimum threshold of 15–25 cases is exceeded [117,118,119]. In the minimally invasive era, a significant reduction of the conversion rate and an increase in the lymph node yield was reported after the 10th case [120]. Moreover, comparing well-trained laparoscopic surgeons working in high- and low-volume hospitals, perioperative outcomes were not influenced, underlining that hospital volume is not a decisive factor [121].

In Japan, the National Clinical Database (NCD) was established in 2010 with the aim of recording all procedures performed by national surgeons. From this project, data on 11,300,000 Japanese patients with gastric cancer were extracted to discuss how surgical and hospital volume impact mortality following surgery for gastric cancer [110]. Interestingly, Iwatsuki M et al. disclosed a strong impact of hospital and surgeon volume on mortality and morbidity rates [110,112]. Particularly, dividing surgeon volume into four groups, S1 (0–2 cases per year), S2 (3–9 cases), S3 (10–25 cases), and S4 (>26 cases), the operative mortality rate after a total gastrectomy decreased from 2.5% in S1 to 0.6% in S4. By contrast, after proper statistical analysis adjusted by risk model variables (demographic factors, preoperative functional status, pre-existing comorbidities, operative factors, and preoperative laboratory data), only hospital volume showed a crucial role in improving outcomes compared with the surgeon volume. In other words, surgeons with low volumes could obtain lower morbidity and mortality rates compared to surgeons with high volumes and worse results. 

Urbach DR et al. assumed that low-volume surgeons may have excellent outcomes because of experience or because they performed a high volume of similar operations requiring similar technical skills [122]. Interestingly, the best postoperative outcomes were obtained by high-volume surgeons in high-volume hospitals, followed by low-volume surgeons in high-volume hospitals [123]. These results may influence surgical training programs and the centralization of advanced surgical procedures. 

However, a more precise standardization of surgical training is needed through dedicated fellowships or the establishment of a minimum skill–volume load for performing certain surgical procedures. If no doubt exists that the accreditation of hospitals improves surgical quality and safety, surgeons’ accreditation programs are currently lacking. The ESSO Core Curriculum, since its conception in 2013 by ESSO, the European Society for Radiotherapy and Oncology (ESTRO), and the European Society of Medical Oncology (ESMO), has served as a guidance document for surgical oncologists to obtain the level of knowledge needed both for surgical oncology practice but also for the European Board of Surgery Qualification (EBSQ) in surgical oncology. In October 2021, an update on ESSO Core Curriculum was published [124], with the aim to give the candidate an idea of expectations and areas for in-depth study, in addition to the practical requirements to “*permit flexibility to suit the needs of the different regions of the world with their inherently diverse sociocultural, financial and cultural differences*”—Audisio R. In this way, the paradox of having a particular hospital accredited to perform several complex procedures without having qualified accredited surgeons can be avoided. It is time to shift from the pursuit of high-volume to high-quality centers.

On the other hand, the annual surgeon activity can only represent a surrogate marker for medical care quality [125], since it may not cover the complexity of this issue consisting of hospital volume, specialization, and mentorship opportunities [114]. Quality of care, in fact, consists of more than the performance of a single surgeon. Organizational effectiveness, perioperative care, anesthesia, ICU staffing, the experience of the nursery staff, nutritional evaluation, comprehensive geriatric assessment [85], and collaboration between different disciplines all contribute to the outcomes of the performed procedure [25]. 

### 3.3. Textbook Outcome

In 2017 the Dutch Upper Gastrointestinal Cancer Audit (DUCA) group designed the Textbook Outcome (TO), a multidimensional scale that provides an ideal route after esophagogastric cancer surgery [126]. It comprises ten perioperative quality-of-care parameters:

(1) Complete, potentially curative, resection as judged by the surgeon at the time of surgery;
(2) No intraoperative complication;
(3) Negative resection margin;
(4) Greater than 15 lymph nodes sampled;
(5) No severe postoperative complications (Clavien–Dindo grade II or higher);
(6) No re-intervention (surgical, endoscopic, or radiological) ≤30 days after surgery;
(7) No unplanned ICU or medium-care unit (MCU) admission ≤30 days after surgery;
(8) Duration of stay not exceeding 21 days;
(9) No 30-day readmission;
(10) No 30-day mortality following surgery.

They demonstrated that the quality of surgical care for patients with gastric cancer is multidimensional, and it is possible to generate supplementary information when different outcome parameters are combined into a single comprehensive outcome measure. TO was achieved in 48.6% (569/1172 patients) of patients with gastric cancer, resulting in a good match of 30-day postoperative mortality (5.5%) and severe postoperative complications (11.7%) when compared with other contemporary results [25,127]. 

In van der Kaaij’s RT series, TO was associated with long-term overall survival (OS) after surgery for gastric cancer. Patients with a TO had 1-, 2-, and 3-year overall survival rates of 85%, 70%, and 64%, respectively, versus 64%, 49%, and 42% for patients with no TO, respectively. Good patient selection, well-performed surgery, and optimal postoperative care can ensure a rapid discharge, optimize long-term outcomes, and reduce costs for the healthcare system. Interestingly, the DUCA group achieved TO in 23% of patients in hospitals performing 0 to 19 gastrectomies per year, 29% in hospitals performing 20 to 39 gastrectomies per year, and 27% in hospitals performing more than 40 gastrectomies per year [128,129].

The next update of the Population Registry of Esophageal and Stomach Tumors of Ontario (PRESTO) group did not include radical resection according to the surgeon and intraoperative complications (previously not unambiguously differentiated from postoperative complications) [130]. Overall, the new TO definition included eight points in total and was achieved in 24.6% of patients with gastric cancer. First, the proportion achieving TO varied significantly by year of surgery and displayed a significant and positive trend (20.3% in 2004 and 29.3% in 2015, *p* < 0.001). Secondly, surgeons and hospitals were ranked into quintiles (Q): surgeon Q1 performing 0 gastrectomies per year to surgeon Q5 performing 3.5–9.5 gastrectomies per year, and hospital Q1 with 0–2 volume per year to hospital Q5 with 12–22 procedures. TO was achieved in a higher percentage of patients treated in the highest volume hospitals compared to the lowest volume ones (Hospital Q5 23.5% vs. Q1 16.2%), while similar TO results were obtained by the highest and lowest volume surgeons (Surgeon Q5 24.0% vs. Q1 20.8%). This discrepancy was due to the adequate lymph node sampling rate, the lower rate of unplanned ICU admissions, and lesser 30-day mortality. However, neither TO nor 30-day postoperative morbidity, readmission, and mortality were associated with surgeon or hospital volumes.

In 2022, the same group concluded that achieving TO is strongly associated with improved long-term survival in 1836 gastric cancer patients, with a 41% reduction in 3-year mortality (*p* < 0.001) [131].

According to Levy J et al., the volume–outcome relationship is analogous to practice-makes-perfect, whereas “*perfect practice makes perfect*” may be more effective [130]. Future policies should be focused more on meeting quality parameters than on absolute volume.

Anyway, new scientific evidence is shedding light on the grey zones of the management of gastric cancer, focusing researchers’ efforts on new outcomes. This is the premise for setting a new TO for gastric cancer.

## 4. European Recommendations

Vonlanthen R, on behalf of members of the European Surgical Association (ESA), presented 12 recommendations for future development strategies in centralization:

(1) The definition should be based on disease (i.e., pancreatic cancer) or on organ systems (i.e., complex HPB diseases) rather than a procedure (i.e., esophagectomy or pancreatectomy);
(2) Planning is based on a minimum number of cases per center and well distributed among the various regions, taking into account the demographic and cultural specificities of a country;
(3) Planning should include at least two centers per country to secure choice and competition (except for small countries and very rare diseases);
(4) Adequate resources must be ensured with an appropriate assessment of the available infrastructure and personnel;
(5) Centers must offer fully functioning multidisciplinary teams (MDTs) of specialists able to deal with all aspects of the diseases throughout the year;
(6) Adequate care and follow-up are ensured by the presence of the centers connected to a network of hospitals;
(7) Centralization specifications must be legally applied for adherence to the specifications applied locally and regionally and for private and non-private hospitals;
(8) The centralization process must be accompanied by mainstream media activities to ensure adequate public awareness;
(9) Centers are required to have an externally verified database, to be actively involved in clinical studies (including RCTs), and should be supported to contribute to laboratory research;
(10) Quality control must be accompanied by international benchmark comparative studies;
(11) Equal accessibility to centralized healthcare should be monitored;
(12) Centers are expected to participate in surgical training and provide specialized training, as well as rotation of general surgeons [132].

Furthermore, an obvious gap between regulations for centralization and implementation was registered, especially in the private sector compared to publicly “subsidized” hospitals. Overall, obstacles to centralization could be recognized at different levels: (a) healthcare provider (insufficient infrastructure, lack of specialized personnel, long waiting time), (b) patient (resistance to longer travel distance, to cultural and language changes, lack of awareness of better outcome), (c) payer, i.e., insurance, government (concerns from increased cost or charges), (d) political level (political decision are not enforced, regional interests outweigh centralization policies, legal divergences, conflict of interest, overwhelming bureaucracy, lack of specialization boards and of board recognition among countries) [132].

There are at least two possible solutions to the fragmentation of the care process and to patient trends and geographical needs consequent to an increase in centralization: on one hand, the implementation of surgical fellowships and training of medical staff in higher volume hospitals and younger surgeons working in lowest volume centers; on the other hand, the creation of hospital and territorial clinical and oncological networks, to ensure standard and multidisciplinary care [133]

## 5. Italian Perspective

How centralization should be implemented remains a controversy and in many countries, the focus lies on the centralization of complex surgical procedures.

The Italian National Health Care Outcomes Program (*Programma Nazionale Esiti*, PNE, https://pne.agenas.it, accessed on 14 March 2023), a tool developed by the National Agency for Regional Health Services (AGENAS), evaluates the outcome measurements in Italian hospitals. In 2021, PNE recorded a total of 5075 gastrectomies performed in Italian hospitals, with a higher prevalence of cases treated in hospitals in the north of the country. According to the volume of interventions, 274 (54.9%) institutions registered more than 5 gastrectomies per year; of these, only 60 hospitals (21.9%) performed more than 20 gastrectomies per year.

Overall postoperative 30-day mortality was 5.62%. Low-volume centers’ mortality rate ranged from 10 to 20%, while in high-volume centers a mortality rate of 3–5% was registered. The threshold of low adherence to quality standards was accordingly set at 10%.

Since there are no strict regulations due to the absence of a formal policy of centralization, gastric cancer surgery is still executed anywhere in Italy. Nowadays, a referral pathway for cancer patients has been introduced only in several Italian regions, i.e., Campania, Lazio, Liguria, Lombardia, Toscana, Piemonte, Veneto, Valle d’Aosta, with the vast majority organized according to a hub and spoke model. As a result, differently from other countries, an Italian agreement about the minimum volume standards of gastrectomies has yet to be established and attempts for its definition come from scientific societies, such as the Italian Society of Surgery (SIC) and the Italian Society of Surgical Oncology (SICO).

Lorenzon L et al. reported that 40.4% of the hospitals treating patients with gastric cancer performed less than five procedures/year in 2018. Classifying institutions by volume, the mean mortality was 7.7% in institutions performing 1–3 resections, compared to 4.7% in the highest volume institutions, 17–127 resections/year (*p* < 0.001) [134]. Moreover, the authors noted that the number of gastrectomies in each Italian province does not reflect the actual number of gastric cancers diagnosed in the same zone and that the pattern of health-related travels usually follows a south-to-north trend.

The Italian Research Group on Gastric Cancer (GIRCG) is implementing an Italian centralization policy for gastric cancer surgery, acting on the national healthcare system and with the support of the scientific community. Its recent guidelines can be a useful tool to address physicians in managing gastric cancer patients [3]. Based on the principles set forth in these statements, physicians will adhere to the best, internationally accepted, effective standard of care.

## 6. Conclusions

Interpretations of studies on this topic require caution. Hospital and surgeon volumes act as a proxy measure and a surrogate of technical and non-technical items to be identified and evaluated in both low- and high-volume centers. It is time to drop Birkmeyer’s aphorism “*the more I do, the better I do*” [135,136], to share “*perfect practice make perfect*” [130]. Careful selection of outcomes is essential for decision-makers, clinical professionals, and patients to improve clinical practice, guide health policy, and drive healthcare choices. The textbook outcome is a novel quality measure, reflecting the “ideal” surgical outcome.

Although the centralization of complex surgical procedures is totally sensible, since it is potentially associated with a higher quality of care, clear criteria are still lacking on what, where, and whom to centralize. The ESA recommendations may serve as a basis for discussion to improve healthcare in surgical oncology.

Emphasis on multidisciplinary evaluations and clinical decision-making such as pre-habilitation, standardized clinical pathways, and perioperative noninvasive management has improved the hospital care of patients with gastric cancer. High-volume centers boast the cooperation of healthcare professionals and services to support the provision of care.

The definition of centers of excellence equally distributed across the country, well-organized multidisciplinary networks, and centralization of high-risk procedures, as well as advanced training for new generations, accreditation of surgeons, and monitoring of surgical performance, should be the priorities.

## Data Availability

Not applicable.
